# *Toxoplasma gondii* tachyzoite-extract acts as a potent immunomodulator against allergic sensitization and airway inflammation

**DOI:** 10.1038/s41598-017-15663-4

**Published:** 2017-11-09

**Authors:** Mirjana Drinić, Angelika Wagner, Priya Sarate, Christian Zwicker, Elke Korb, Gerhard Loupal, Roman Peschke, Anja Joachim, Ursula Wiedermann, Irma Schabussova

**Affiliations:** 1 Institute of Specific Prophylaxis and Tropical Medicine, Medical University of Vienna, Vienna, Austria; 2 Institute of Pathology and Forensic Veterinary Medicine, Department of Pathobiology, University of Veterinary Medicine Vienna, Vienna, Austria; 3 Institute of Parasitology, Department of Pathobiology, University of Veterinary Medicine Vienna, Vienna, Austria

## Abstract

Epidemiological and experimental studies have shown an inverse relationship between infections with certain parasites and a reduced incidence of allergic diseases. We and others have shown that infection with *Toxoplasma gondii* prevents the development of allergy in mice. To establish whether this beneficial effect could be recapitulated by soluble products of this parasite, we tested an extract derived from *T*. *gondii* tachyzoites. Immunization of BALB/c mice with tachyzoites lysate antigen (TLA) elicited mixed Th1/Th2 responses. When TLA was applied together with the sensitizing ovalbumin (OVA), the development of allergic airway inflammation was reduced, with decreased airway hyperresponsiveness associated with reduced peribronchial and perivascular cellular infiltration, reduced production of OVA-specific Th2 cytokines in lungs and spleens and reduced levels of serum OVA-specific IgG1 as well as IgE-dependent basophil degranulation. Of note, TLA retained its immunomodulatory properties, inducing high levels of IL-6, TNFα, IL-10 and IL-12p70 in bone marrow-derived dendritic cells after heat-inactivation or proteinase K-treatment for disruption of proteins, but not after sodium metaperiodate-treatment that degrades carbohydrate structures, suggesting that carbohydrates may play a role in immunomodulatory properties of TLA. Here we show that extracts derived from parasites may replicate the benefits of parasitic infection, offering new therapies for immune-mediated disorders.

## Introduction

The prevalence of allergic, inflammatory and autoimmune diseases has been increasing in industrialized countries in the last few decades^1^. This trend has been correlated with lifestyle changes, such as increase in hygiene measures, the wide use of antibiotics, vaccinations, as well as reduced exposure to different infectious agents, such as bacteria, protozoa and helminths^2–4^. This correlation has received significant interest, resulting in formulation of the hygiene hypothesis^5^ and subsequently, in many epidemiological and experimental studies. For example, infection with helminth parasites, like *Heligmosomoides polygyrus* ^6^, *Litomosoides sigmodontis* ^7^ or *Trichinella spiralis* ^8^ have been shown to reduce allergic airway inflammation in several murine models. Importantly, beneficial effects of infections with certain helminths on chronic inflammatory conditions have also been observed in clinical trials^9–14^. However, the introduction of potentially pathogenic parasites as a treatment strategy raises several concerns regarding the safety of such an application. Therefore, there is an interest in identifying parasite-derived molecules to treat immune-mediated diseases or to use them as adjuvants with immunomodulatory effects in future allergy vaccines. In this respect, parasitic products were already tested and their beneficial effects on experimental allergy have been shown^15,16^.

*Toxoplasma gondii* is an obligatory intracellular protozoan parasite with wide range of hosts, including humans and with approximately 30% prevalence worldwide^17^. The infection with *T*. *gondii* develops upon ingestion of oocysts, the parasitic stage shed in feces of cats that contaminate soil, food or water, or through the consumption of raw or undercooked meat containing tissue cysts^18^. Once ingested, the oocysts rupture in the gut and release sporozoites into the lumen. Sporozoites infect enterocytes and differentiate into tachyzoites, which are highly proliferative, mobile, and rapidly disseminate throughout the body, inducing an acute immune response^17,19^. Although acute infection is usually asymptomatic in immunocompetent host, in rare occasions it may result in fever and lymphadenopathy followed by chronic infection. In immunosuppressed patients and in fetuses it can cause severe disease, with frequent manifestations in the eye or the central nervous system^18,20^. Interestingly, epidemiological studies suggested inverse associations between *T*. *gondii* seropositivity and allergic sensitization^21–23^. Along these lines, we and others have previously shown that experimental infection with *T*. *gondii* suppressed allergic airway inflammation in mouse models^24,25^. Furthermore, we have shown that not only the infection with live *T*. *gondii*, but also the treatment with crude extract derived from *T*. *gondii* oocysts (OLA) can reduce the development of allergic responses in a mouse model of birch pollen allergy^26^. However, the collection of *T*. *gondii* oocysts for OLA preparation is linked with several obstacles, such as necessity of passage of the parasite through the feline host, low parasite yield or possible contamination with bacterial components, especially endotoxin, or host-derived material. On the contrary, tachyzoites can be cultured *in vitro* in Vero cells, allowing the collection of *T*. *gondii* compounds in high quantity without the risk of contamination. Most importantly, there is no loss of immunogenic properties through continuous passages^27–29^. This culturing method thus offers several biological, ethical and economic benefits over protocols for isolation of other developmental stages, such as oocysts.

The aim of this study was to evaluate immunomodulatory properties of tachyzoites lysate antigen (TLA) and to establish whether experimental allergy could be prevented by this extract. TLA is a mixture of proteins and carbohydrates^30–32^ and the identification of specific suppressive factors in TLA may lead to development of novel therapeutic applications to control immune-mediated inflammatory diseases.

## Results

### TLA triggers the production of pro- and anti-inflammatory cytokines in naïve splenocytes *in vitro*

**Figure 1:**
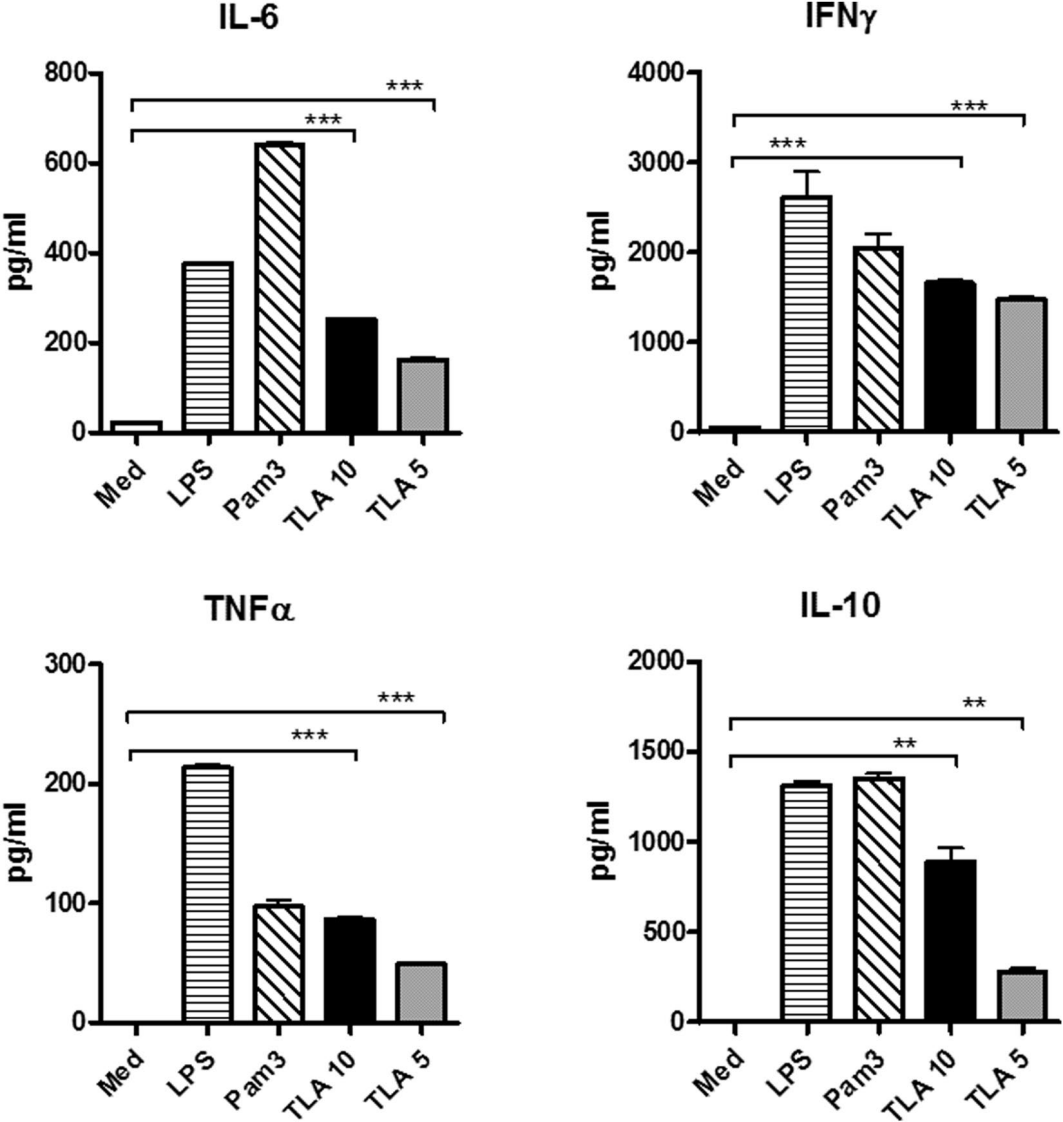
Cytokine production of TLA-stimulated splenocytes. Splenocytes from naïve mice were cultured with media only (Med), 10 and 5 µg/ml TLA for 72 h. Ultra-pure lipopolysaccharide from *E*. *coli* (LPS; 1 µg/ml) and Pam3CSK4 (Pam3; 1 µg/ml) were used as positive controls. Levels of cytokines in culture supernatants were determined by ELISA. Three replicate cultures with cells from individual mice were measured. All data are representative of at least three independent experiments performed using different batches of TLA. Error bars show mean ± SEM. Results of Student´s t test: **P < 0.01, ***P < 0.001.

To determine the immunostimulatory capacity of TLA *in vitro*, splenocytes from naïve BALB/c mice were cultured for three days with 5 and 10 µg TLA. In comparison to cells stimulated with medium only, TLA-stimulated cells induced significant levels of pro-inflammatory cytokines such as IL-6, IFNγ, TNFα as well as the anti-inflammatory cytokine IL-10 (Fig. 1).

### Immunization with TLA elicits mixed Th1/Th2 humoral and cellular immune responses

**Figure 2:**
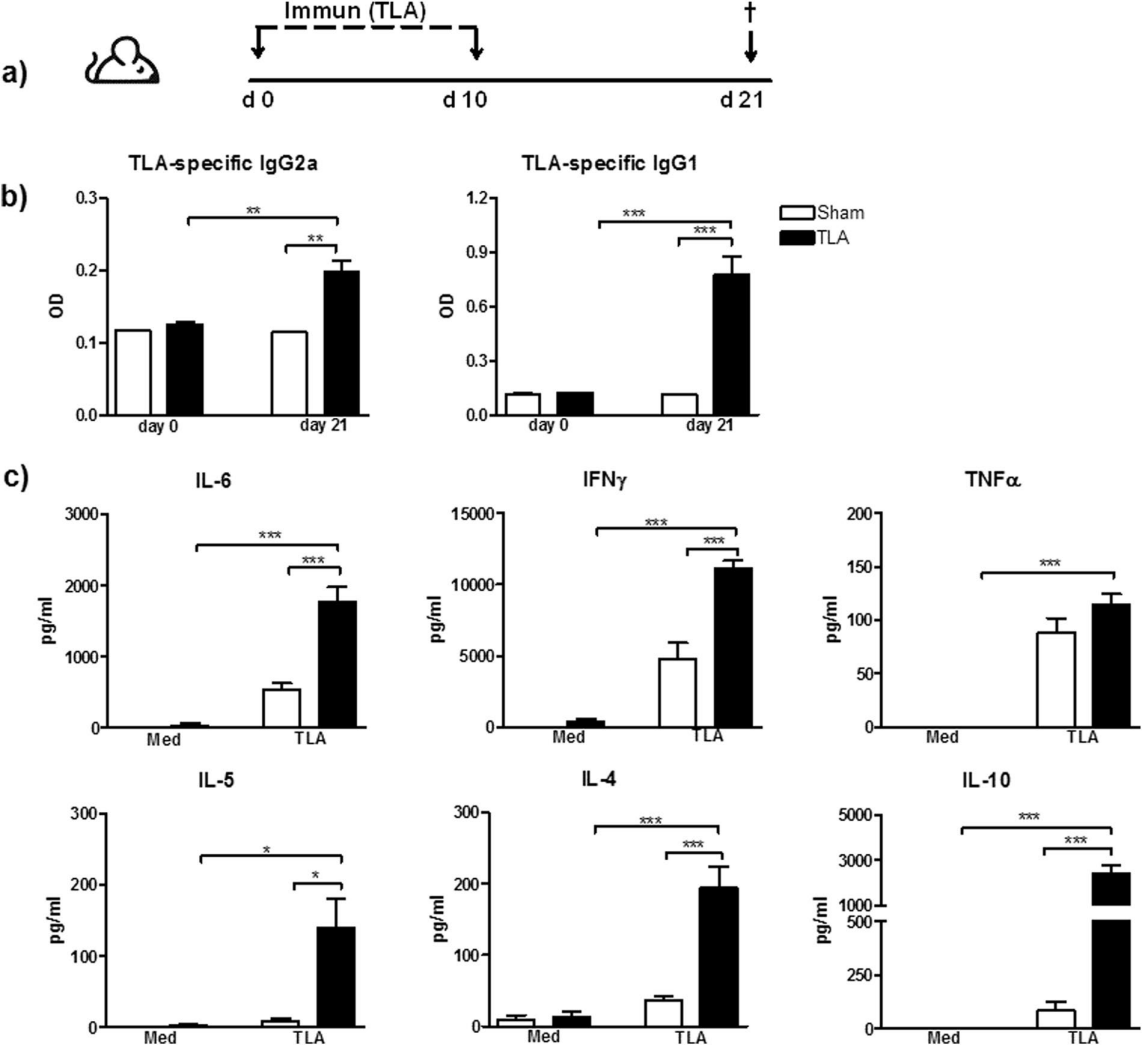
Antibody production and recall responses in mice immunized with TLA. Mice were immunized intraperitoneally with 10 µg TLA in alum on days 0 and 10 (TLA group). Controls received PBS in alum (Sham group) (**a**). Levels of TLA-specific serum antibodies collected on day 0 and day 21 were determined by ELISA (**b**). Spleens were collected at day 21 and single cell suspensions (5 × 10^6^ cells/ml) were cultured in the presence of medium only (Med) or 5 µg/ml TLA for 72 h. Levels of cytokines in culture supernatants were determined by ELISA (**c**). Data are representative of three experiments (n = 5 mice per group). Results of Student´s t test: *P < 0.05, **P < 0.01, ***P < 0.001.

To test humoral and cellular immune responses induced by TLA *in vivo*, BALB/c mice were immunized with TLA in alum or sham-treated with PBS in alum (Fig. 2a). Levels of TLA-specific antibodies in serum were measured on day 0 and 21. TLA-immunization led to significant production of TLA-specific IgG2a (Th1-associated isotype), as well as TLA-specific IgG1 (Th2-associated isotype) when compared to levels in pre-immune serum collected on day 0 and to levels in serum of sham-treated mice collected on day 21 (Fig. 2b). However, TLA-specific levels of another Th2-associated antibody, IgE, remained unchanged (measured OD for the TLA group was 0.17 ± 0.005 and 0.18 ± 0.007 on day 0 and 21, respectively; P = 0.2481). Restimulation of spleen cells from TLA-immunized mice with TLA *ex vivo* led to increased levels of Th1-related cytokines IL-6, IFNγ and TNFα, as well as Th2 cytokines IL-5 and IL-4 and regulatory IL-10 compared to cultures incubated with medium only. Similarly, stimulation of splenocytes from TLA-immunized mice with TLA induced higher production of most of these cytokines in comparison to levels detected in spleen cell cultures from TLA-stimulated sham-treated mice (Fig. 2c).

### TLA reduces AHR and prevents allergic inflammation in lungs

**Figure 3:**
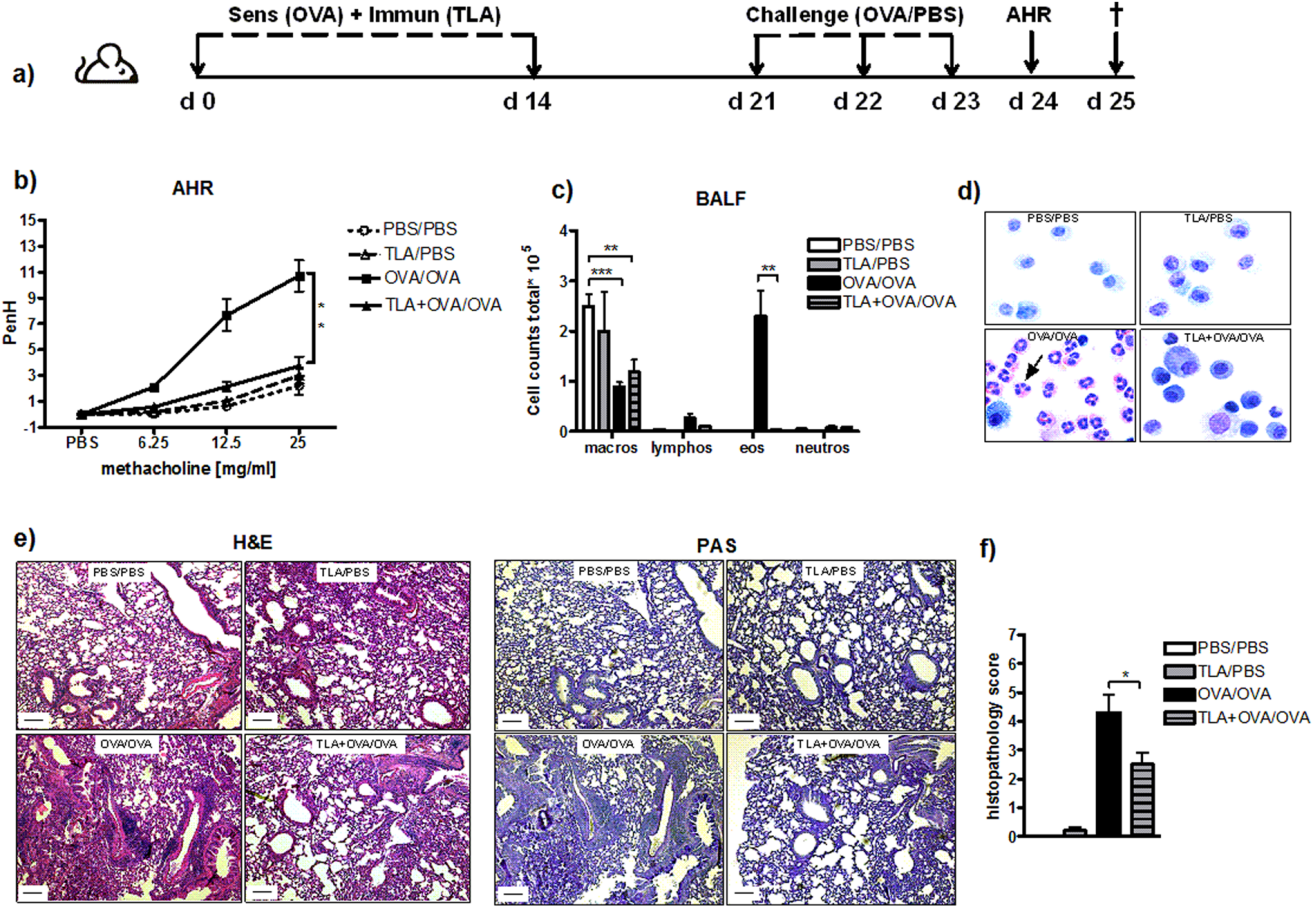
Effect of TLA on the development of allergic airway inflammation. Mice were immunized intraperitoneally with 50 µg of TLA admixed to 10 µg of ovalbumin in alum (TLA + OVA/OVA group) on day 0 and 14 and intranasal challenge with 100 µg of OVA was given at days 21–23. Control groups were immunized with 10 µg of ovalbumin in alum (OVA/OVA), 50 µg TLA in alum (TLA/PBS) and PBS in alum (PBS/PBS) and challenged with OVA or PBS (**a**). Airway hyperresponsiveness (AHR) was assessed in the response to inhalation of increasing doses of methacholine on day 24 (**b**). Number of differential cells in bronchoalveolar lavage (BALF) (**c**) and representative cytospins of BALF stained with haematoxylin and eosin (H&E; 100x magnification; arrow indicates eosinophil) (**d**). Representative lung tissue sections stained with H&E and Periodic Acid Schiff (PAS; 40x magnification; scale bars, 100 µm) (**e**). Mean gross lung histopathology score (**f**). Data are representative of three experiments (n = 5 mice per group). Results of ANOVA test: *P < 0.05, **P < 0.01, ***P < 0.001.

**Figure 4:**
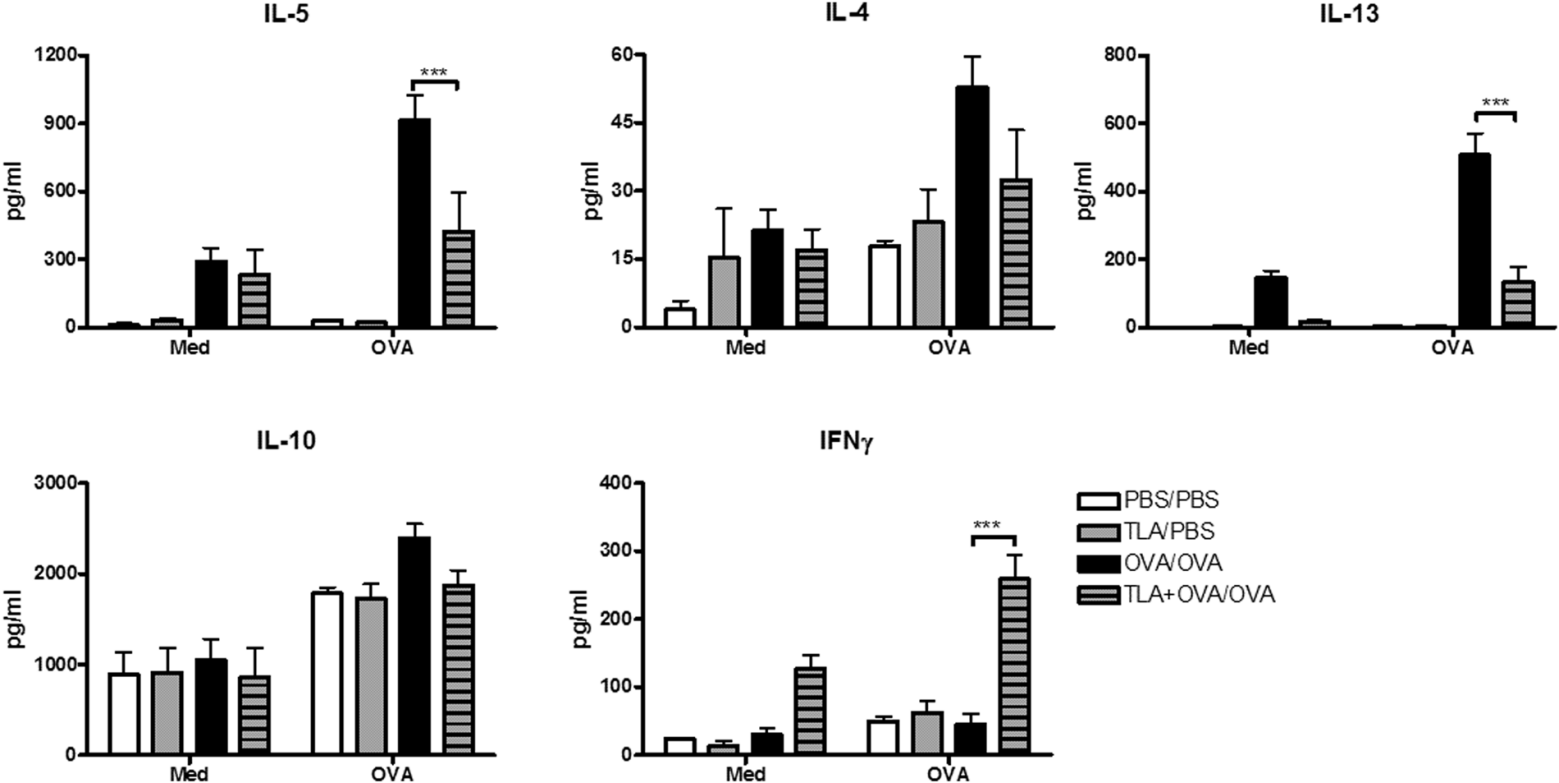
Effect of TLA on the production of cytokines in lungs. Mice were treated as indicated in Fig. 3a. Lungs were collected on day 25 and single cell suspensions (5 × 10^6^ cells/ml) were cultured in the presence of medium (Med) or 50 µg/ml OVA for 72 h. Levels of cytokines in culture supernatants were assessed by ELISA. Data are representative of three experiments (n = 5 mice per group). Results of ANOVA test: ***P < 0.001.

To investigate whether TLA can modulate allergic lung inflammation, we used a mouse model of systemic sensitization and intranasal challenge with OVA. Mice were immunized i.p. with alum-adjuvanted OVA in the presence (TLA + OVA/OVA group) or absence (OVA/OVA group) of TLA. Control groups were immunized with PBS (PBS/PBS group) or TLA (TLA/PBS group) in alum only and challenged with PBS as indicated in Fig. 3a. A dose-dependent increase in AHR to methacholine, a nonspecific bronchoconstrictor, is one of the characteristics of allergic asthma^33^. As expected, intranasal challenge with OVA led to increased AHR in OVA/OVA mice in comparison to PBS/PBS mice, as demonstrated by increased levels of methacholine-induced PenH (Fig. 3b). Furthermore, OVA challenge led to increased numbers of eosinophils in BALF and increased numbers of inflammatory cells and goblet cell hyperplasia in lungs in comparison to PBS/PBS mice (Fig. 3c–f). Intraperitoneal treatment of sensitized mice with TLA (TLA + OVA/OVA) significantly suppressed the development of AHR compared to OVA/OVA mice, resulting in PenH values similar to those seen in negative controls (Fig. 3b). Reduced AHR was associated with reduced eosinophils in BALF (Fig. 3c,d) and reduced infiltration of inflammatory cells into perivascular and peribronchiolar connective tissues (Fig. 3e; H&E staining). In parallel, TLA reduced goblet cell hyperplasia in lungs in comparison to OVA/OVA group (Fig. 3e; PAS staining). Immunization with TLA without OVA-sensitization and challenge (TLA/PBS) led to a similar outcome as sham-treatment (PBS/PBS) (Fig. 3b–f). Stimulation of lung cell cultures of OVA/OVA mice with OVA *ex vivo* led to high levels of IL-5, IL-4, IL-13, and IL-10 (Fig. 4). Lung cells of TLA + OVA/OVA mice exhibited reduced IL-5 and IL-13, but increased levels of IFNγ in response to OVA (Fig. 4). Levels of IL-4 and IL-10 were reduced as well, but the difference did not reach statistical significance (Fig. 4).

### TLA inhibits the development of Th2-type allergen-specific immune responses

**Figure 5:**
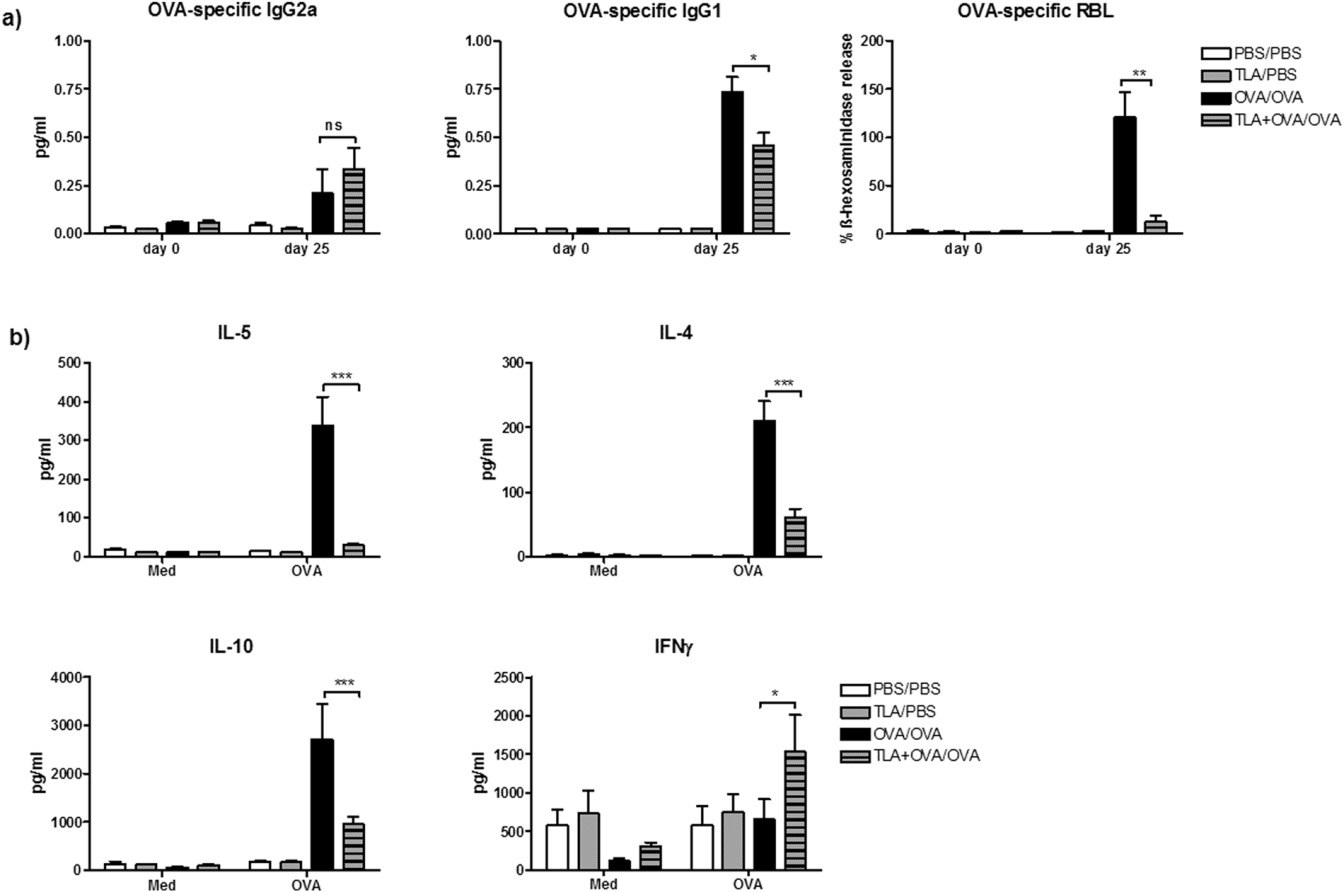
Effect of TLA on the development of systemic immune responses. Mice were treated as indicated in Fig. 3a. Serum and spleens were collected on day 25. Levels of OVA-specific IgG2a and IgG1 were determined by ELISA. Degranulation of rat basophil leukaemia cells (RBL) by cross-linking of the FceRI-bound IgE with allergen (0.3 µg/ml OVA) was measured by the production of β-hexosaminidase (**a**). Levels of cytokines in supernatants from spleen cells cultured with media only (Med) or 50 µg/ml OVA for 72 h was measured by ELISA (**b)**. Data are representative of three experiments (n = 5 mice per group). Results of ANOVA test: *P < 0.05, **P < 0.01, ***P < 0.001.

In order to determine whether TLA could affect also the induction of allergen-specific humoral responses, we measured the levels of OVA-specific antibodies in serum. Samples were collected before sensitization (d 0) and after the sensitization and challenge on the sacrifice (d 25). As shown in Fig. 5a, mice sensitized and challenged with OVA (OVA/OVA) exhibited increased levels of specific antibodies in sera collected on day 25 in comparison to levels in samples collected on day 0 (Fig. 5a). Although TLA-treatment in TLA + OVA/OVA group had no impact on the levels of OVA-specific IgG2a (Fig. 5a), it markedly reduced levels of IgG1 as well as the IgE-dependent basophil degranulation in the RBL assay (Fig. 5a). Furthermore, we aimed to exclude the possibility that certain soluble factors in sera of TLA + OVA/OVA mice interfere with the basophil degranulation in RBL assay. Therefore, RBL cells were preincubated with sera of TLA-immunized mice prior to incubation with sera of OVA-sensitized. The results indicate, that reduced levels of β-hexosaminidase in cultures treated with sera from TLA + OVA/OVA mice in comparison to levels obtained by incubation with sera from OVA/OVA mice is not caused by soluble factors present in the sera of TLA-treated mice (Supplementary Fig. S1). The levels of OVA-specific antibodies in TLA/PBS were comparable with levels detected in PBS/PBS mice (Fig. 5a). Additionally, restimulation of spleen cell cultures of TLA + OVA/OVA mice with OVA led to reduced production of Th2 cytokines IL-5 and IL-4, as well as of regulatory IL-10 in comparison to cultures of OVA/OVA mice. However, TLA-treatment of OVA-sensitized mice led to increased levels of IFNγ in TLA + OVA/OVA mice in comparison to mice in OVA/OVA group (Fig. 5b). In these experiments, PBS/PBS and OVA/OVA groups were evaluated as negative and positive controls, respectively, to determine the effect of co-application of TLA and sensitizing allergen on the development of airway inflammation. Comparison to PBS/PBS group enables to resolve the potential consequence of TLA-treatment on immunological parameters in the lungs. However, in order to determine the baseline for measurement of TLA-induced effects (interference during sensitization) on allergen-induced airway inflammation, additionally, we measured humoral and cellular immune responses in non-sensitized and OVA-challenged mice and compared them to PBS/PBS and OVA/OVA groups. Here, we could clearly show that non-sensitized OVA-challenged mice exhibit comparable levels of AHR, eosinophils in BAL, inflammatory infiltrates, Th2 cytokines in the lungs and OVA-specific antibodies in serum, when compared to PBS/PBS mice. Furthermore, all measured parameters were markedly different when compared to levels observed in OVA/OVA mice (Supplementary Fig. S2-4).

### Immunostimulatory potential of TLA is not impaired by heat-inactivation

**Figure 6:**
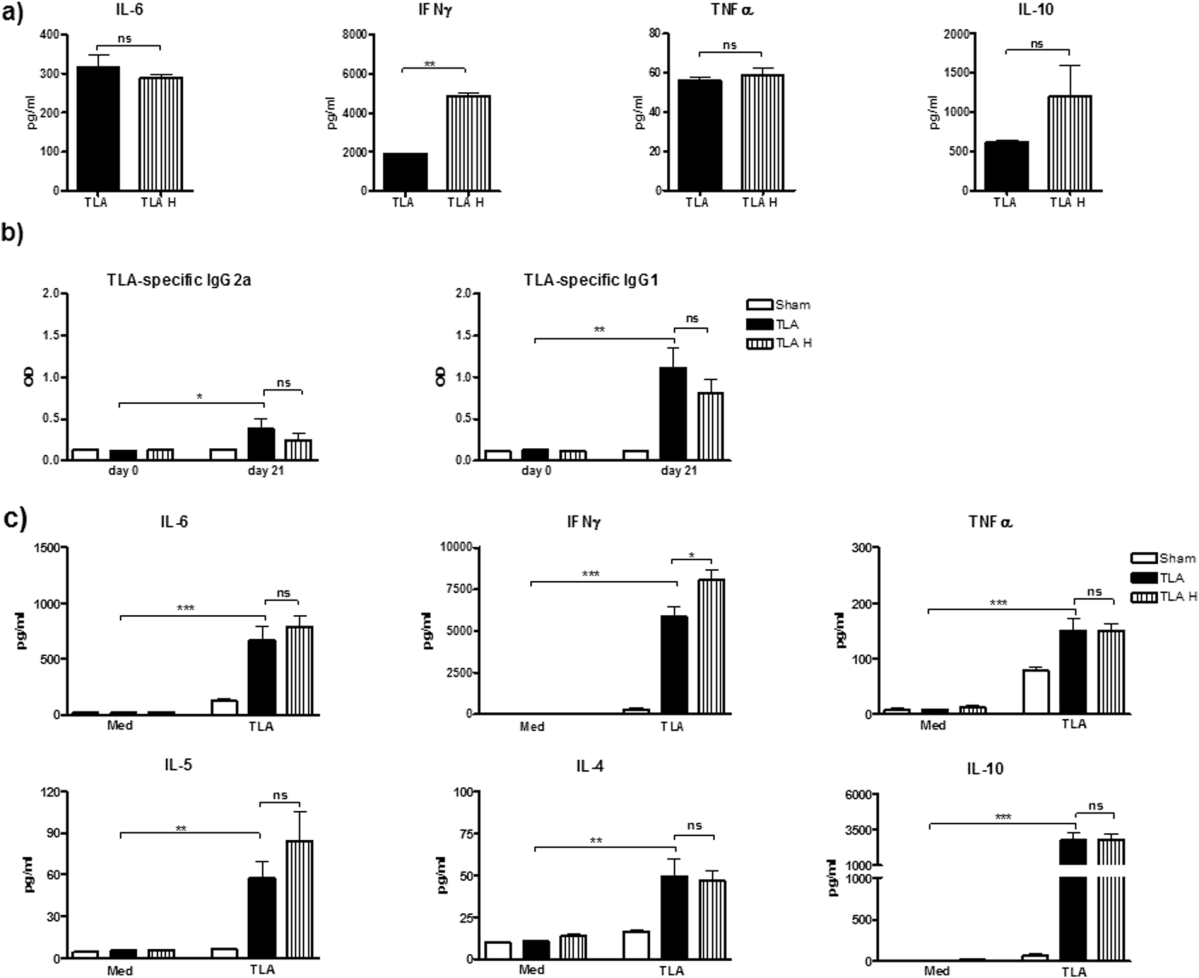
Impact of heat-treatment on the immunomodulatory properties of TLA. Splenocytes of naïve mice were cultured with 5 µg of native TLA (TLA) or heat-inactivated TLA (TLA H) for 72 h (**a**). Mice were immunized intraperitoneally with 10 µg TLA in alum (TLA group) or 10 µg of heat-inactivated TLA (TLA H group) on days 0 and 10. Controls received PBS in alum (Sham group). Serum and spleens were collected on day 21. TLA-specific IgG2a and IgG1 in serum were assessed by ELISA (**b**). Spleen cell cultures were restimulated with 5 µg/ml TLA or with medium only (Med) and incubated for 72 h. Levels of cytokines in culture supernatants were determined by ELISA (**c**). Data are representative of three experiments (n = 5 mice per group). Results of ANOVA test: *P < 0.05, **P < 0.01, ***P < 0.001.

To investigate whether TLA-components responsible for immunomodulation are heat-stable, TLA was heated at 96 °C for 15 min to denature proteins (TLA H) and tested *in vitro* and *in vivo*. Stimulation of splenocytes from naïve mice with TLA or TLA H led to comparable production of IL-6, TNFα and IL-10. TLA H was more potent in the induction of IFNγ in comparison to native TLA (Fig. 6a). In order to investigate the effect of heat-inactivation *in vivo*, mice were immunized twice with TLA and TLA H and TLA-specific humoral and cellular responses between both groups (TLA and TLA H) were compared (Fig. 6b,c). As shown in Fig. 6b, levels of TLA-specific antibodies on day 21 were comparable between both experimental groups. Stimulation of spleen cells of mice immunized with TLA or TLA H with native TLA led to comparable levels of IL-6, TNFα, IL-5, IL-4 and IL-10 (Fig. 6c). Interestingly, immunization with the heat-inactivated TLA led to an increased production of IFNγ in stimulated splenocytes compared to immunization with the native TLA.

### TLA loses its immunostimulatory potential upon deglycosylation

**Figure 7:**
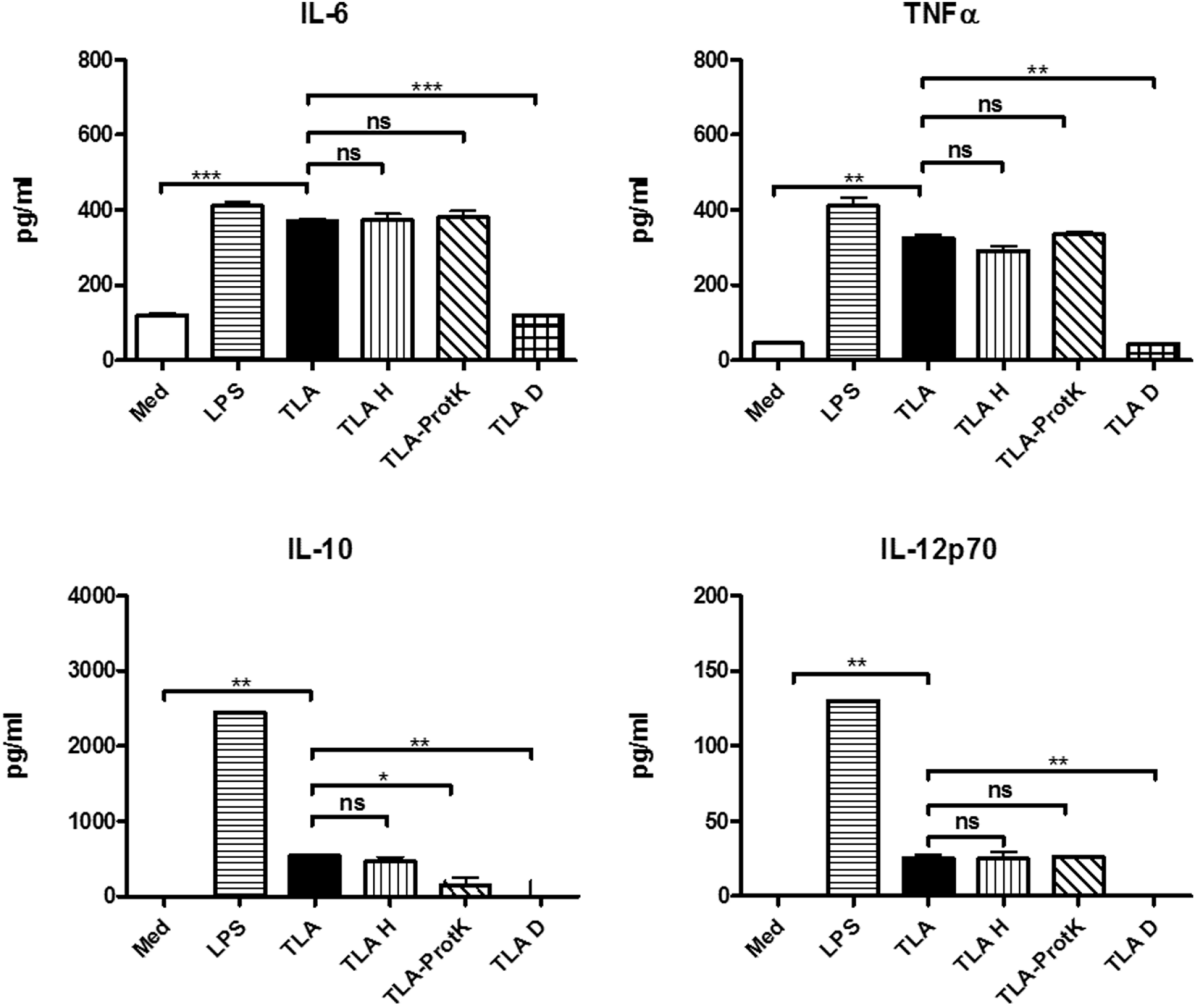
*In vitro* stimulation of bone marrow-derived dendritic cells with heat-inactivated, proteinase K-treated and deglycosylated TLA. Bone marrow-derived dendritic cells (BMDC) were cultured with media only (Med), 5 µg/ml TLA (TLA), heat-inactivated TLA (TLA H), TLA digested with proteinase K (TLA-ProtK), or deglycosylated TLA (TLA D), and LPS (1 µg/ml) for 24 h. Levels of cytokines in culture supernatants were determined by ELISA. All data are representative of at least two independent experiments. Results of Student´s t test: *P < 0.05, **P < 0.01, ***P < 0.001.

Our results suggest that proteins are not the key players involved in the TLA-induced immunomodulation, therefore TLA was exposed to different biochemical treatments and tested *in vitro*. In order to confirm the hypothesis that not the proteins but rather carbohydrates are involved in the immunomodulation, TLA was treated with proteinase K (TLA-ProtK) to digest proteins into peptides or with sodium metaperiodate (TLA D), to modify glycan moieties. Sodium metaperiodate treatment leads to destruction of carbohydrate integrity through alteration of three-dimensional structures of the molecules^34^. By using a panel of nine biotinylated lectins in an ELISA assay, we could show that TLA contains wide range of carbohydrate moieties, which were removed by sodium metaperiodate treatment (Supplementary Fig. S5). TLA-ProtK, TLA D, as well as TLA and TLA H, were used for *in vitro* stimulation of BMDC, which were isolated from naïve BALB/c mice. Levels of cytokines IL-6, TNFα, and IL-12p70 did not differ between TLA, TLA H and TLA-ProtK and were significantly increased compared to medium levels. Levels of IL-10 were reduced in TLA-ProtK-stimulated cells in comparison to levels observed in cultures with native TLA. Interestingly, TLA D failed to stimulate cytokine production in BMDC, which indicates that glycan moieties might be responsible for the immunomodulatory effect of TLA (Fig. 7).

## Discussion

Studies in both humans and animal models demonstrated that certain parasitic infections modulate the host immune responses, which is often associated with protection against allergic diseases^6,8,35^. For example, respiratory allergies are less frequent among individuals infected with the protozoan parasite *T*. *gondii* ^21,23^. In experimental settings we have previously shown that *T*. *gondii* infection, as well as inactivated extract of *T*. *gondii* oocysts prevented the development of airway inflammation in a mouse model of birch pollen allergy^24,26^. Similarly, Fenoy *et al*. have shown that *T*. *gondii* infection reduced the airway inflammation in a mouse model of OVA-induced allergy^25,36^. In the present study we tested immunomodulatory properties of an extract derived from *T*. *gondii* tachyzoites, *in vitro* and *in vivo* in naïve mice and in OVA-allergy model.

Although the immune response to acute infection with *T*. *gondii* in humans and mice is shifted towards Th1 type, which is mandatory to control parasite replication^24,37,38^, we observed that immunization of mice by i.p. injection of TLA in alum induced mixed Th1/Th2 humoral and cellular immune responses. We detected substantial levels of *T*. *gondii*-specific serum IgG2a, a Th1-associated isotype as well as IgG1, a Th2-associated isotype, in samples collected after two immunizations at sacrifice. Similarly, high levels of both Th1 and Th2 cytokines, such as IFNγ and TNFα, or IL-4 and IL-5, respectively, were detected in TLA-stimulated splenocytes. These results are in agreement with our previous findings, as well as with the study by Costa-Silva *et al*., who described that immunization of mice with *T*. *gondii*-derived products induced mixed humoral and cellular Th1 and Th2 responses^39,40^.

Furthermore, we could show that TLA application in combination with allergic sensitization and challenge suppressed allergic immune response to OVA, demonstrated by reduced airway hyperresponsivness, reduced influx of eosinophils, peribronchial and perivascular infiltration of inflammatory cells, as well as Th2 cytokines in lungs compared to sensitized controls. In parallel, levels of Th2 cytokines in OVA-restimulated splenocytes as well as Th2-associated specific serum antibody IgG1 were reduced in TLA-treated mice in comparison to sham-treated sensitized controls. The potential of TLA to reduce Th2 responses has been documented by Liesenfeld *et al*. in a mouse model of infection with *Nippostrongylus brasiliensis* ^37^. The authors showed that administration of TLA prior to *N*. *brasiliensis* infection reduced levels of total IgE in serum and numbers of eosinophils in the periphery. However, the suppressive effect on Th2 responses was short-lived. The experimental setup in our study does not allow us to investigate whether the anti-allergic effects of TLA are long-lasting, therefore, follow-up experiments will focus on this aspect.

Here we have shown that TLA-treatment led to increased levels of IFNγ in OVA-restimulated lung and spleen cell cultures. Similar effect was observed previously in allergic mice with *T*. *gondii* infection^24,36^. Although high levels of IFNγ has been associated with exacerbated symptoms of asthma in humans and mice^41^, the importance of this cytokine in allergy prevention was demonstrated for example in the study by Brand *et al*. who could show that the farm-derived bacterial strain *Acinetobacter Iwoffii* F78, prevented the development of allergy in IFNγ-dependent manner^42^. Also, induction of IFNγ has proven to be crucial in the prevention of house dust mite allergy^43^.

On the other hand, a regulatory cytokine IL-10 has been shown to play an important role in parasite-induced suppression of allergy^44–46^. TLA induced high levels of IL-10 in *vitro* in spleen cell cultures derived from naïve or TLA-immunized mice and in cultures with BMDC. However, in TLA-treated allergic mice this cytokine was not detectable in OVA-stimulated lung and spleen cell cultures. This suggests that IL-10 is not a key cytokine involved in TLA-induced allergy prevention and this is in agreement with findings by Fenoy *et al*., who could show that *T*. *gondii*-induced suppression of allergy is IL-10-independent^47^. We have previously shown that decreased levels of antigen-specific IL-10 in cell cultures could be explained by increased uptake via upregulated IL-10 receptor^48^. However, the cellular source of IFNγ and its role, as well as the exact role of IL-10 in allergy-suppression by TLA still remains to be investigated.

In order to investigate the nature of molecules responsible for immunomodulatory effects, TLA was heat-inactivated or treated with proteinase K to elucidate the relevance of proteins, or treated with sodium metaperiodate to clarify the role of glycans. We have demonstrated that deglycosylated TLA failed to induce IL-6, TNFα, IL-10 or IL-12p70 production in stimulated dendritic cells, whereas interference with protein activities did not influence stimulatory properties of TLA, suggesting that heat-stable carbohydrates might play an important role in parasite-host interaction and immunomodulatory effect of TLA.

Parasite-derived glycans have been shown to play an important role in immunomodulation. For example, glycoconjugates from *Trichuris suis*, *Fasciola hepatica* or the major immunogenic glycan element of schistosomes, Lewis^x^, have been shown to induce activation of antigen-presenting cells and participate in modulation of host immunity by interacting with C-type lectins^49–51^. Of interest, it has been shown that glycosylation is a common modification of *T*. *gondii* proteins^52^. In tachyzoites, enzymes involved in *O*-glycosylation are constitutively expressed and novel *O*-linked glycosylated proteins have been identified^53^. Also the presence of *N*-glycosylation pathway in tachyzoites of *T*. *gondii* has been demonstrated and it has been shown that several key proteins involved in invasion and motility are N-linked glycoproteins^30,54^. Our data indicate that glycans of *T*. *gondii* tachyzoites have a role in the immunomodulation and studies on detailed characterization of the glycosylation pattern of TLA are in progress.

Taken together, we could show that TLA exhibits strong immunostimulatory properties *in vitro* and *in vivo*, inducing mixed Th1/Th2 profile in BALB/c mice. Furthermore, co-application of TLA with the allergen reduced allergic sensitization and airway inflammation in a mouse model of OVA allergy. The fact that the immunostimulatory potential of TLA is lost upon deglycosylation indicates that its immunomodulatory effects are glycan-mediated. Our findings contribute to the understanding of host-parasite interactions and may pave the way for the design of novel well-tolerated immunotherapies for human immune-mediated disorders.

## Materials and Methods

### Animals

Female BALB/c mice aged 6–8 weeks were purchased from Charles River (Sulzfeld, DE) and kept under conventional housing conditions. All experiments were approved by the Animal Experimentation Committee of Medical University of Vienna and the Austrian Federal Ministry of Education, Science and Culture and all methods were performed according to their guidelines and regulations.

### Parasites

*T*. *gondii* tachyzoites (strain S-48) were cultured in Vero cells (ATCC Number CCL-81, LGC Standards GmbH, DE) for 4 days in RPMI-1640 medium (Sigma-Aldrich, USA) supplemented with 4% FBS (HyClone^®^, GE Healthcare, UK) and 1% L-Glutamine-Pen/Strep × 100 (Sigma-Aldrich) under 5% CO_2_ and at 37 °C. Tachyzoites were purified from Vero cells by discontinuous Percoll^®^ (GE Healthcare Biosciences AB, SE) density gradient centrifugation (9,000 × *g*, 15 min) and resuspended in PBS. Counting was performed in a Bürker-Türk-chamber (Hecht-Assistent^®^, DE) and 2 × 10^9^ *T*. *gondii* tachyzoites per ml PBS were stored at −80 °C.

### Preparation of TLA

TLA was prepared by three freeze-thaw cycles in liquid nitrogen, followed by sonication (10 × 30 s at 2 min intervals at 4 °C) in the presence of protease inhibitors (Complete^®^ Protease Inhibitor Cocktail tablets; Roche, DE) and subsequently centrifuged at 10,000 × *g* (60 min at 4 °C). Supernatant was collected, sterile filtered (22 µm Millex^®^GV Filter Unit, Merck Milipore, IRL) and protein concentration was measured with a BCA Protein Assay Reagent kit (Pierce Peribo, USA). Endotoxin levels in TLA were determined by Limulus Amoebocyte Lysate (LAL) (Endpoint Chromogenic LAL Assay; Lonza LTD, CH) and they were below 0.1 EU in 1 µg of extract. TLA was stored at −80 °C until use.

### Heat-inactivation, proteinase K and periodate treatment

Heat-inactivation of TLA was achieved by incubation at 96 °C for 15 min (TLA H). Furthermore, TLA was enzymatically treated with 1 mg/ml proteinase K (QIAGEN GmbH, DE) at 37 °C overnight, followed by 20 min incubation at 96 °C in order to inactivate the enzyme (TLA-ProtK). Sodium metaperiodate-mediated modification of glycan moieties in TLA (TLA D) was performed as follows. 450 µg TLA was treated with 50 µl 100 mM sodium metaperiodate (Sigma-Aldrich) to yield final concentration of 10 mM, at pH 4.5 for 45 min at room temperature (RT) in the dark. The oxidation reaction was stopped with 100 µl of sodium borohydride (Merck) at a final concentration of 50 mM, at pH 4.5 for 30 min at RT in the dark. Excess salt was removed by exchanging reaction buffer with PBS by using desalting columns (Zeba Spin Desalting Columns, 7 K MWCO, Pierce Biotechnology, Thermoscientific, USA) and the protein concentration was assessed as above.

### Stimulation of splenocytes and dendritic cells

Single cell suspensions were prepared from spleens of naïve BALB/c mice and resuspended in complete media (RPMI-1640 supplemented with 10% heat-inactivated FCS, 2 mM L-glutamine, 2 mM mercaptoethanol, 100 µg/ml gentamicin; Sigma-Aldrich). Cells (5 × 10^6^/ml) were incubated with 10 and 5 µg/ml TLA, 5 µg/ml TLA H, synthetic triacylated lipopeptide (Pam3CSK; 1 µg/ml; Invivogen, USA) and ultra-pure lipopolysaccharide from *E*. *coli* (LPS; 1 µg/ml; Invivogen) for 72 h.

Bone marrow precursor cells were isolated from murine femurs and tibias of naïve female BALB/c mice and cultured in complete media supplemented with GM-CSF (20 ng/ml; Peprotech, USA) as described previously^16^. On day 8, bone marrow-derived dendritic cells (BMDC) (1 × 10^6^/ml) were incubated with media only, 5 µg/ml TLA, TLA H, TLA-ProtK, TLA D or LPS (1 µg/ml) for 24 h. Both splenocytes and BMDC cultures were kept in the incubator at 37 °C and 5% CO_2_. Supernatants were collected and kept at −20 °C until further use. Detection of cytokines IL-6, IL-10, IL-12p70, IFNγ and TNFα in supernatants was performed by ELISA (Ready-Set-Go Kit, eBioscience, USA) according to the manufacturer´s instructions.

### Immunization with TLA and heat-inactivated TLA

Mice were immunized intraperitoneally with 10 µg TLA in 140 µl alum (Alu-Gel-S Suspension, Serva Electrophoresis, DE) (TLA group), 10 µg heat-inactivated TLA in alum (TLA H group) or sham treated with PBS in alum (Sham group) on days 0 and 10. Blood samples were collected one day before the first immunization (day −1) and at the end of the experiment (day 21). Serum obtained after blood coagulation and centrifugation for 10 min at 1,500 × *g* was stored at −20 °C for further analysis. Levels of TLA-specific IgG1 and IgG2a antibodies were measured by ELISA. Briefly, microtiter plates (Nunc, DK) were coated with 4 µg/ml of TLA in coating buffer (0.1 M carbonate-bicarbonate buffer, pH 8.4) overnight at 4 °C. Coated plates were incubated with blocking buffer (PBS/0.05%Tween/1% BSA) for 6 h at RT. Plates were washed and incubated with diluted serum samples overnight at 4 °C. Sera were diluted 1:1000 for IgG1, 1:500 for IgG2a and 1:10 for IgE. On the following day, plates were washed and incubated with rat-anti-mouse IgG1, IgG2a and IgE (1:500; Pharmingen, USA) for 6 h at RT. Plates were washed and incubated with horseradish peroxidase-conjugated mouse-anti-rat IgG (1:2000; Jackson ImmunoResearch Laboratories Inc., USA) for 1 h at 37 °C and then 1 h at 4 °C. Plates were washed, incubated with chromogenic substrate (1 mM ABTS in 70 mM citric-phosphate buffer, pH 4.2; Sigma-Aldrich). Absorbance was measured at 405 nm on SparkControl Magellan plate reader (Tecan GmbH, AT). At sacrifice (d 21), spleens were collected and single cell suspensions were prepared and cultured (5 × 10^6^/ml) with 5 µg/ml TLA or media alone at 37 °C for 72 h. Supernatants were collected and levels of cytokines measured by ELISA as indicated above.

### Mouse model of OVA-induced airway inflammation

Mice were immunized intraperitoneally with the mixture of 50 µg TLA and 10 µg OVA (grade V; Sigma-Aldrich) in 90 µl alum on days 0 and 14; and then challenged three times intranasally with 100 µg OVA in a final volume of 30 µl on days 21–23 (TLA + OVA/OVA group). Before each challenge, mice were anesthetized by 5% isoflurane (Isocare; 100% w/v; Inhalation vapour, Animalcare Ltd, UK) in UniVet Porta anaesthesia machine (Groppler Medizintechnik, DE) with the airflow set at 3 L/min. Control groups were sensitized with 10 µg OVA and challenged with OVA (OVA/OVA group); treated with 50 µg TLA and challenged with 30 µl PBS (TLA/PBS group) or sham treated with PBS and challenged with PBS (PBS/PBS group). Mice were terminally anesthetized and organs were excised on day 25. In order to prepare single cell suspension, spleens were forced through a cell strainer and hemolyzed for 1 min in 3 ml hemolysis buffer (150 mM ammonium chloride, 10 mM potassium bicarbonate and 0.1 mM EDTA). Lung cell isolation was performed as previously described^55^. Briefly, lungs were excised and minced in 6 ml serum-free RPMI-1640 media containing Liberase TL (0.5 mg/ml; Roche, DE) and DNase I (0.5 mg/ml; Sigma-Aldrich), incubated for 45 min at 37 °C and finally, the remaining tissue was forced through a 70 µm cell strainer (Falcon, Corning Inc., USA). Spleen and lung single cell cultures (5 × 10^6^/ml) were restimulated with media and 50 µg/ml endotoxin-free OVA (EndoGrade Ovalbumin, Hyglos GmbH, DE) for 72 h. Supernatants were collected and cytokines were measured by ELISA as described above. Levels of OVA-specific IgG1 and IgG2a were measured by ELISA. Microtiter plates were coated with 5 µg/ml OVA in coating buffer and ELISA was carried out as above.

### Rat basophil leukaemia cell-based assay

Rat basophil leukaemia (RBL) cell mediator release assay was performed as previously described^56^. Briefly, RBL-2H3 (4 × 10^5^/ml) cells were incubated with diluted sera (1:300) and degranulation was induced by adding 0.3 µg/ml OVA (grade V; Sigma-Aldrich) in Tyrode´s buffer (134 mM NaCl, 12 mM NaHCO_3_, 2.9 mM KCl, 0.34 mM Na_2_HPO_4_, 1 mM MgCl_2_, 10 mM HEPES, pH 7.4; Sigma-Aldrich). Fluorescence was measured at λ_ex_:360 nm/λ_em_:465 nm using a fluorescence plate reader. Results represent the percentage of total ß-hexosaminidase release after addition of 1% Triton X-100.

### Assessment of airway hyperresponsiveness

Airway hyperresponsiveness (AHR) was measured 24 h after the last intranasal challenge by whole body plethysmography (DSI, Buxco Electronics Inc., USA) in conscious unrestrained animals in response to increasing doses (0–25 mg/ml) of aerosolized methacholine (acetyl-β-methyl-choline chloride, Sigma-Aldrich). The baseline was established in response to PBS inhalation, and airway resistance was determined by calculating dimensionless parameter enhanced pause (PenH) as previously described^57^.

### Differential cell counts in bronchoalveolar lavage fluid

Lungs were lavaged with 2 × 1 ml PBS (137 mM NaCl, 2.7 mM KCl, 4.3 mM Na_2_HPO_4_, 1.47 mM KH_2_PO_4_; pH 7.4) and collected bronchoalveolar lavage fluid (BALF) was centrifuged at 300 × *g* for 5 min at 4 °C. For differential cell counts, the cell pellet was resuspended and spun onto microscopic slides at 800 × *g* for 3 min (4 × 10^4^ cells; Shandon Cytospin^®^, Shandon Southern Instruments, UK), air-dried and subsequently stained with haematoxylin and eosin (H&E; Hemacolor^®^, Merck, DE). Altogether, 200 macrophages, lymphocytes, eosinophils and neutrophils cells per slide were counted under the light microscope (Nikon Eclipse; 100x magnification).

### Lung histology

Excised lung tissue was fixed with 7.5% formaldehyde-PBS and paraffin-embedded. 5 µm tick sections were stained with H&E and periodic acid-Schiff (PAS) stain. Histological pathology score was evaluated using light microscopy according to the method adapted from Zaiss *et al*.^58^: (i) perivascular and peribronchiolar inflammation (grade: 0 = no changes; 1 = few perivascular and peribronchiolar inflammatory cells; 2 = moderate numbers of cell cuffs on several perivascular and peribronchiolar sites; 3 = large number of diffuse infiltrated cell cuffs), (ii) presence of leukocytes in alveolar spaces (grade: 0 = no cells; 1 = 2–4 cells; 3 = 4 – 10 cells; 3 = more than 10 cells), and (iii) number of PAS positive cells per 50 counted bronchoalveolar epithelial cells (0 = no cells; 1 = <12; 2 = 12–25; 3 =  > 25). The histopathological score is expressed as a sum of single scores.

### Statistical analysis

Data were statistically analyzed by GraphPad Prism software (Graph Pad Software, USA) utilizing unpaired Student’s *t*-test and two-way ANOVA. All data are shown as mean ± standard error of the mean (SEM) and differences were considered significant at p < 0.05.

## Electronic supplementary material

Supplementary Information
