# Supplementary material for: Toxoplasma gondii tachyzoite-extract acts as a potent immunomodulator against allergic sensitization and airway inflammation
Source: Sci Rep. 2017 Nov 9;7:15211. doi: 10.1038/s41598-017-15663-4 (PMC5680314; doi:10.1038/s41598-017-15663-4)
Supplement: Supplementary file 1 — Supplementary Information [file 41598_2017_15663_MOESM1_ESM.pdf]

Toxoplasma gondii tachyzoite-extract acts as a potent immunomodulator against allergic sensitization and airway inflammation

Mirjana Drinić[1], Angelika Wagner[1], Priya Sarate[1], Christian Zwicker[1], Elke Korb[1], Gerhard Loupal[2], Roman Peschke[3], Anja Joachim[3], Ursula Wiedermann[1] and Irma Schabussova[1*]

[1]Institute of Specific Prophylaxis and Tropical Medicine, Medical University of Vienna, Vienna, Austria

[2]Institute of Pathology and Forensic Veterinary Medicine, Department of Pathobiology, University of Veterinary Medicine Vienna, Vienna, Austria

[3]Institute of Parasitology, Department of Pathobiology, University of Veterinary Medicine Vienna, Vienna, Austria

Figure S1

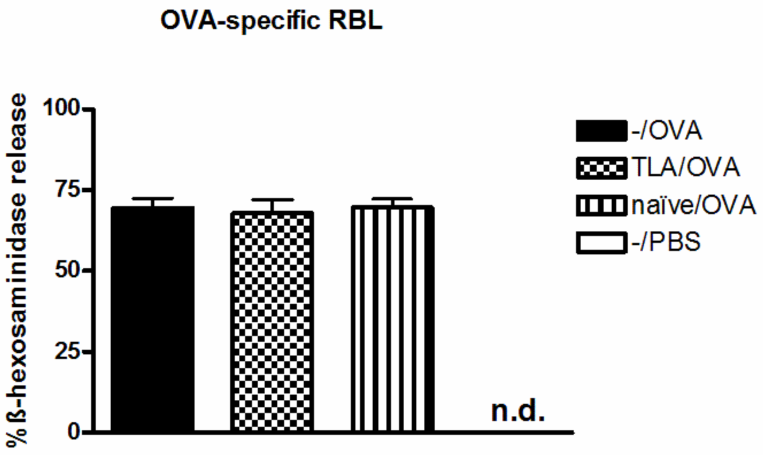

Figure S1. Testing the effect of soluble factors in sera of TLA-immunized mice on basophil degranulation in RBL assay. RBL-2H3 (4 x 10^5/ml) cells were preincubated for 1 h with sera of TLA-immunized mice (TLA/OVA culture; n = 5) or with the sera of naïve mice (naïve/OVA culture; n = 5) followed by incubation with sera of OVA-sensitized mice. Sera from OVA-sensitized and challenged (-/OVA; n = 5) and sham-treated (-/PBS; n = 5) mice were used as controls. Degranulation was induced by 0.3 µg/ml OVA in Tyrode´s buffer and fluorescence was measured at λ_{ex}:360 nm/λ_{em}:465 nm. Results represent the percentage of total ß-hexosaminidase release after addition of 1% Triton X-100. Data are representative of two experiments. n.d = not detected

Figure S2

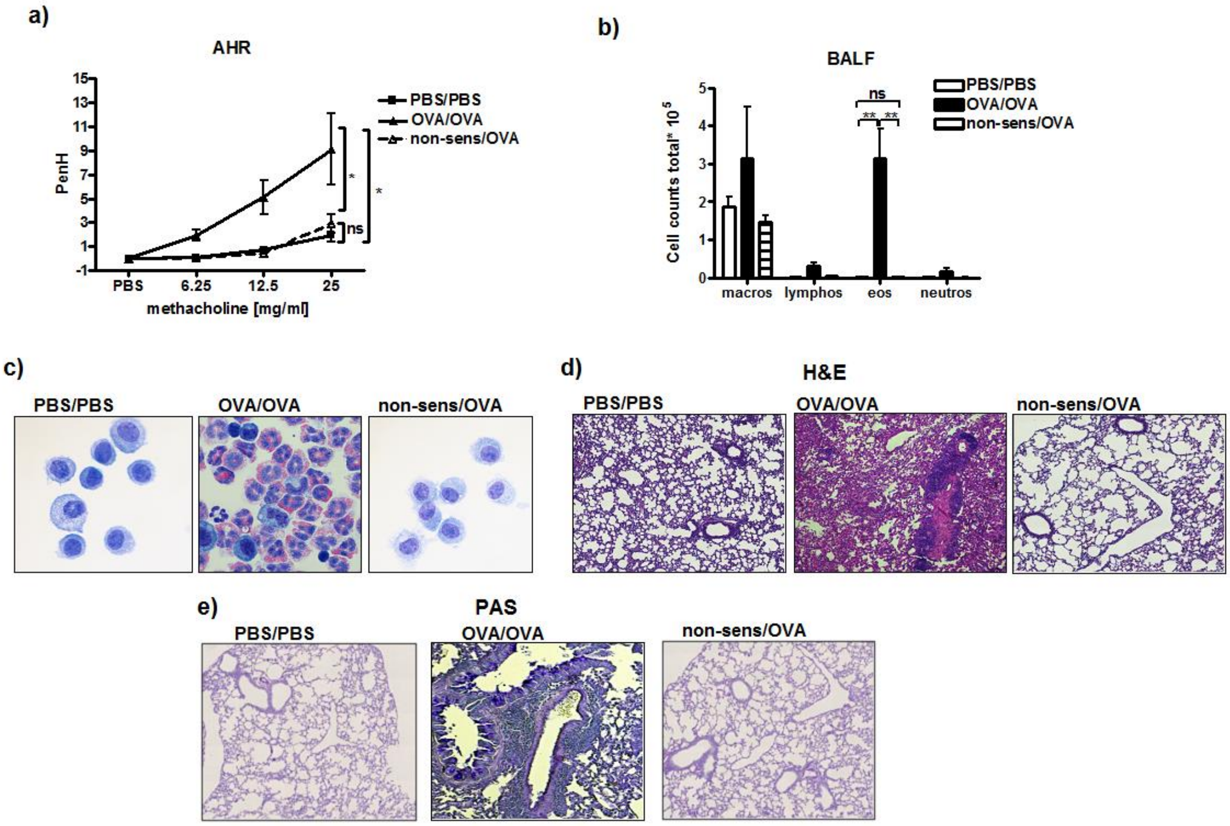

Figure S2. Effect of OVA challenge in non-sensitized mice on development of allergic airway inflammation. PBS/PBS (n = 5) and OVA/OVA (n = 5) mice were sensitized and challenged as described in Fig. 3a. Non-sensitized mice were challenged on 3 consecutive days with 100 µg OVA in PBS (non-sens/OVA; n = 8). Airway hyperresponsiveness (AHR) was assessed in the response to inhalation of increasing doses of methacholine (**a**). Number of differential cells in bronchoalveolar lavage (BALF) (**b**) and representative cytospins of BALF stained with haematoxylin and eosin (H&E; 100 x magnification) (**c**). Representative lung tissue sections stained with H&E (**d**) and Periodic Acid Schiff (PAS) (**e**). Results of ANOVA test: * P < 0.05, ** P < 0.01.

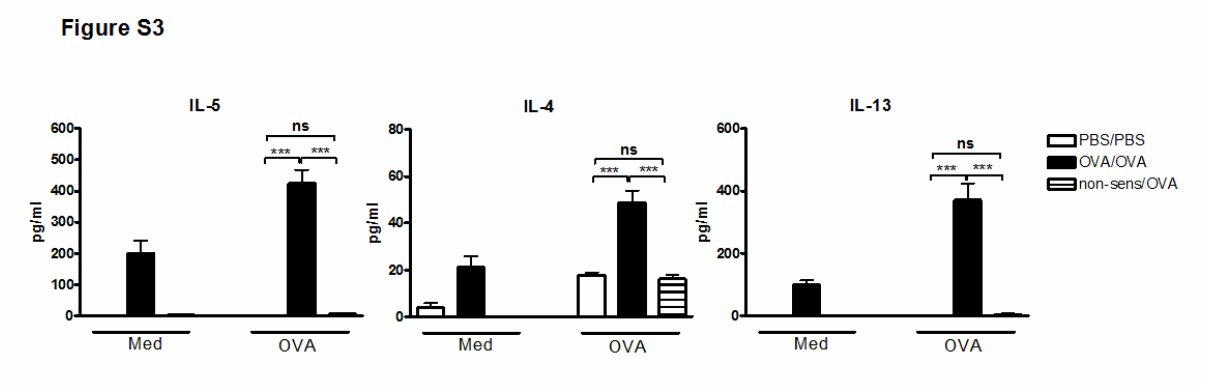

Figure S3. Effect of OVA challenge in non-sensitized mice on the production of cytokines in the lungs. PBS/PBS (n = 5) and OVA/OVA (n = 5) mice were sensitized and challenged as described in Fig. 3a. Non-sensitized mice were challenged on 3 consecutive days with 100 µg OVA in PBS (non-sens/OVA; n = 8). Single cell suspensions of excised lungs (5 x 10^6 cells/ml) were cultured in the presence of medium (Med) or 50 µg/ml OVA for 72 h. Levels of cytokines in culture supernatants were assessed by ELISA. Results of ANOVA test: *** P < 0.001.

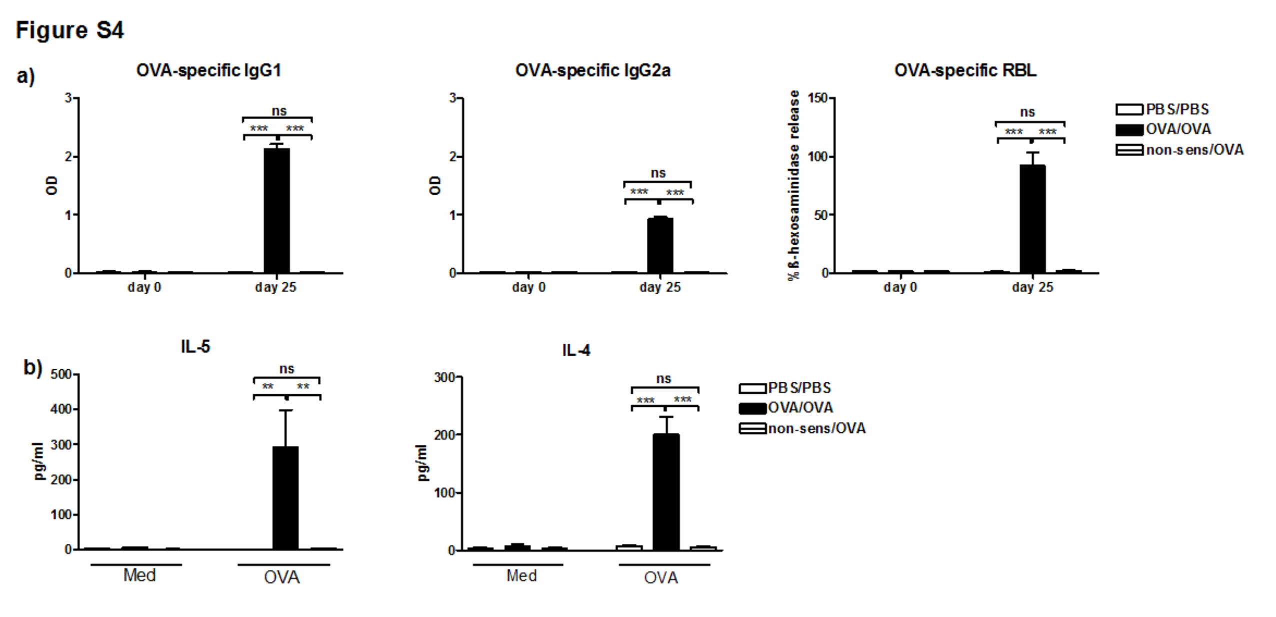

Figure S4. Effect of OVA challenge in non-sensitized mice on the development of systemic immune responses. PBS/PBS (n = 5) and OVA/OVA (n = 5) mice were sensitized and challenged as described in Fig. 3a. Non-sensitized mice were challenged on 3 consecutive days with 100 µg OVA in PBS (non-sens/OVA; n = 8). Levels of OVA-specific IgG2a and IgG1 in serum were determined by ELISA. Functional IgE was measured by OVA-mediated β-hexosaminidase release from rat basophil leukemia cells (RBL) (**a**). Levels of cytokines in supernatants from spleen cells cultured with media only (Med) or 50 µg/ml OVA for 72 h was measured by ELISA (**b**). Results of ANOVA test: * P < 0.05, ** P < 0.01, *** P < 0.001

Figure S5

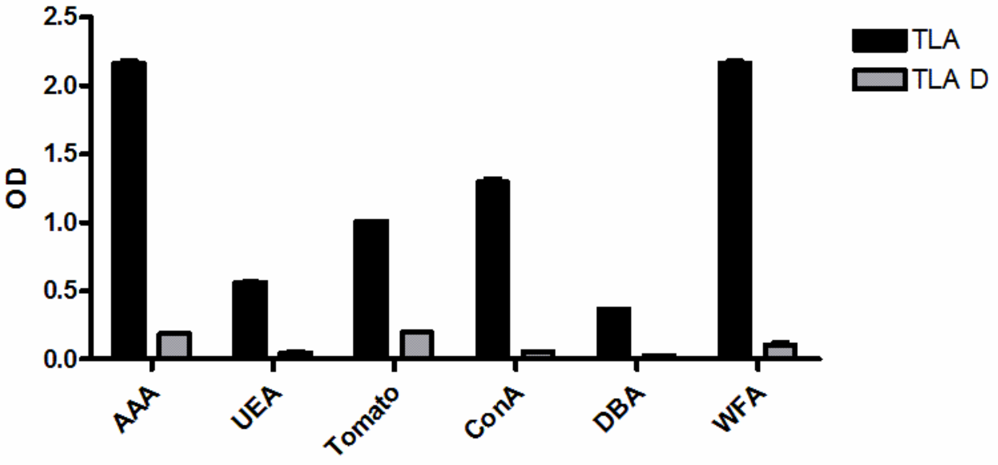

Figure S5. **Enzyme-linked lectin assay with TLA and deglycosylated TLA.** TLA and sodium metaperiodate-treated TLA (TLA D), prepared as described in Fig. 7 were coated in concentration of 15 µg/ml in PBS overnight. Coated plates were incubated with blocking buffer (TBS/3%BSA) for 2 h at RT. Plates were washed and incubated with 1 µg/ml of each biotinylated lectin: AAA (specific for α-Fucose), UEA (specific for Fucose), Tomato (specific for β-N-Acetylglucosamine), ConA (specific for α-Mannose and α-Glucose), DBA (specific for α-N-Acetylgalactosamine), WFA (specific for N-Acetylgalactosamine) for 1 h at RT. Detection was performed with Avidin-HRP and TMB substrate and absorbance was measured at 450 nm.
